# Development of an RNAi-based microalgal larvicide for the control of *Aedes aegypti*

**DOI:** 10.1186/s13071-021-04885-1

**Published:** 2021-08-06

**Authors:** Xiaowen Fei, Yang Zhang, Lili Ding, Sha Xiao, Xiaoqing Xie, Yajun Li, Xiaodong Deng

**Affiliations:** 1 Institute of Tropical Bioscience and Biotechnology, Chinese Academy of Tropical Agricultural Science, Haikou, 571101 China; 2 Department of Biochemistry and Molecular Biology, Hainan Medical College, Haikou, 571101 China; 3 Hainan Provincial Key Laboratory for Functional Components Research and Utilization of Marine Bio-Resources, Haikou, 571101 China

**Keywords:** RNA interference, *Aedes aegypti*, Dengue, *Chlamydomonas*, *Chlorella*

## Abstract

**Background:**

Mosquito-borne diseases affect over half of the human population globally. Multiple studies have shown that chemical insecticides are ineffective because of resistance. Therefore, environmentally safe mosquito population control tools need to be developed. Ribonucleic acid interference (RNAi) is a reverse genetic mechanism recently introduced as a new pest control tool. This technique represents a new class of biorational technology that could combat the increased global incidence of insecticide resistance. The technique has the potential of becoming a critical component of integrated vector control programs.

**Methods:**

A 3-hydroxykynurenine transaminase (3-HKT) RNAi expression plasmid was constructed, generated and transformed into *Chlamydomonas* and *Chlorella* algae. The transgenic algae were then used to feed *Ae. aegypti* mosquito larvae. The feeding experiments were conducted on a small and large scale with 10 and about 300 larvae, respectively. The mortality rate of the larvae was calculated over 30 days. In addition, histological examination of the insect tissues was performed to examine the extent of tissue damage.

**Results:**

The integumentary system and midguts of larvae fed with transgenic *Chlamydomonas* were severely damaged. The mortality rate of the larvae fed with transgenic *Chlamydomonas* ranged from 60 to 100% in small-scale tests. The survival rate of adult mosquitoes was 0.0% in a large-scale feeding experiment when the larvae were fed with transgenic *Chlamydomonas*. Moreover, when the larvae were fed with transgenic *Chlorella*, the mortality rate ranged from 6.7% to 43% compared to that fed wild-type *Chlorella*.

**Conclusions:**

3HKT RNAi transgenic algae are in some scales lethal to *Ae. aegypti*. The findings of this study indicate that technology based on microalgae RNAi may provide a new way to control mosquito populations.

**Graphical Abstract:**

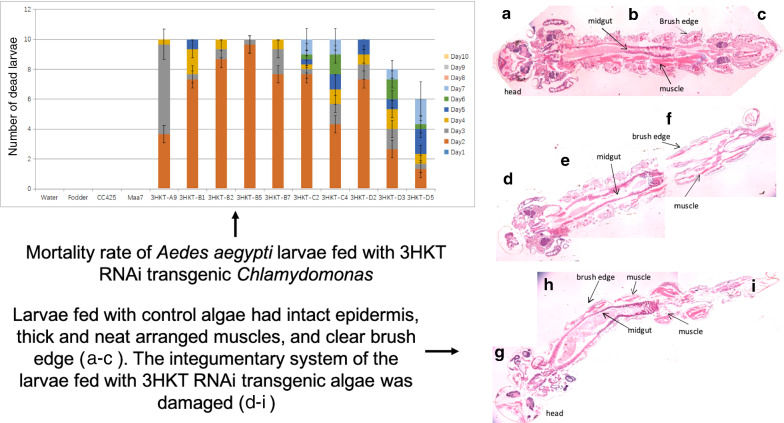

**Supplementary Information:**

The online version contains supplementary material available at 10.1186/s13071-021-04885-1.

## Background

*Aedes* mosquitoes play a significant role in the spread of mosquito-borne diseases. The most severe are dengue fever, Zika virus disease, yellow fever and chikungunya disease [1, 2]. According to WHO, about half of the entire human population is infected with mosquito-borne diseases every year. Since 2007, 128 countries in Asia, Africa, America, Oceania and Europe have reported localized spread of dengue fever. For instance, France and Italy have both reported autochthonous cases of dengue in the last few years [3]. The Zika virus that broke out in 2015 and 2016 has also caused widespread concern because of its ability to cause neonatal abnormalities [4, 5].

Dengue fever is an acute arbovirus disease caused by the dengue virus. *Aedes aegypti* and *Aedes albopictus* are the main vectors of dengue virus. At present, although the CYD-TDV dengue vaccine has been developed, the safety of the vaccine still needs to be tested, and the production cost and output of the vaccine are far from being used on a large scale. Since the vaccine or chemotherapy is not available currently, vector control is the best option to reduce arbovirus burden [6]. Mosquito control is a critical component of human vector-borne disease control. Chemical insecticides are the primary method of controlling the multiplication and spread of mosquitoes, but the excessive use of chemical insecticides has fueled mosquito resistance to almost all known insecticides [7, 8]. Given this, alternative management strategies have been developed, including natural mosquito larvicides obtained from plant extracts, biological control and genetic modification of mosquitoes [9, 10].

At present, there are many reports on the use of RNA interference (RNAi) to successfully silence genes related to various physiological processes in different mosquito species [11–14]. Meanwhile, RNAi has been successfully used to downregulate many target genes in mosquito larvae. This downregulation was confirmed by observing phenotypic changes and performing quantitative reverse transcription-polymerase chain reaction (qRT-PCR) assay. Several methods, such as injection, immersion, nanoparticle and microbial-based techniques, have been employed to deliver double-stranded RNA (dsRNA) to mosquito larvae [15, 16]. The delivery method can be either non-microbiological or microbiological. The non-microbial delivery of RNAi triggers (dsRNA, miRNA, shRNA) is performed via soaking, nanoparticles, injection or osmotic treatment [17–19]. In contrast, microbiological methods employ microorganisms such as bacteria, yeast, algae and viruses to transmit RNAi triggers [20–22]. The application of microbial interfering RNA expression and delivery systems, which facilitate cost-effective RNA propagation, has received substantial attention. Microalgae is a natural food for mosquito larvae and can develop into a dominant species in confined waters. Transgenic microalgae expressing RNAi lethal to mosquitoes can multiply rapidly and control the mosquito population in an area. Kumar et al. [23] explored the feasibility of transgenic *Chlamydomonas reinhardtii* for oral delivery of dsRNA to control *Anopheles stephensi* and minimize the spread of malaria. The tests showed > 30% larval mortality, with the highest mortality of 53% being observed with feeding transgenic algae. In *Aedes* mosquito, 3-hydroxykynurenine transaminase (3-HKT) catalyzes the transamination of 3-hydroxykynurenine (3-HK) to xanthurenic acid (XA) in the tryptophan catabolism pathway [24]. 3-HK is a highly reactive intermediate, which will automatically oxidize under normal physiological conditions to produce reactive oxygen species that can kill insects [24]. Injection of 3-HK into Drosophila is known to induce neuronal apoptosis, resulting in paralysis and death [25]. Therefore, the level of 3-HK is very tightly regulated by rapid conversion to XA by 3-HKT. Thus, silencing the 3HKT gene may lead to the death of *Aedes* mosquitoes. In this study, we designed an experiment to generate dsRNA by constructing an RNAi based vector containing a reverse repeat sequence of the 3-HKT target gene. The vector was transformed into *Chlamydomonas* or *Chlorella* to obtain algal strains lethal to mosquitoes. Considering that the dengue fever epidemic in southern China is mainly transmitted by *Aedes* mosquitoes, we constructed the 3-HKT gene RNAi expression vector targeting *Aedes* mosquitoes. Also, given the large-scale industrialization of *Chlorella*, and its higher survival ability in natural waters, compared to *C. reinhardtii*, we transformed both *Chlamydomonas* and *Chlorella* with the 3HKT RNAi genes.

## Methods

### Mosquito maintenance

The rearing of mosquitoes is described in our previous report [26]. We used *A. aegypti* (Rockefeller strain), a lineage that has been reared continuously in the laboratory for 2 years. The mosquitoes were reared in a colony room maintained at 26 °C and 70–80% relative humidity. Adult insects were maintained in a 10% sucrose solution, and the adult females were fed with chicken blood for egg-laying. The larvae were reared on rat food (Mazuri, PMI).

### Algal strains and culture conditions

The *C. reinhardtii* strain CC425 (cw15 arg2) was purchased from the Chlamy Center at Duke University and grown in Tris-acetate phosphate (TAP) medium supplemented with 250 µg ml^−1^ arginine [27, 28]. Liquid cultures were maintained at 25 °C in a shaker (200 rpm) under continuous light of 150 µmol m^−2^ s^−1^. Strains on TAP-agar plates were incubated at 22 °C under a light intensity of 100 µmol m^−2^ s^−1^ [29]. *Chlorella vulgaris* HOC5 was isolated from Qinglan Bay, Hainan, by our research team and cultured on TAP medium. In this study, it was used as the recipient algae strain. The culture conditions were 22 °C with a light intensity of 100 µmol m^−2^ s^−1^.

### Construction of pMaa7 IR/3HKTIR, an RNAi vector harboring the 3HKT gene

Complementary DNA (cDNA) reverse transcribed from the total RNA of *Ae. aegypti* was used as the template for PCR amplification. The first strand of cDNA was synthesized using SuperScript III Reverse Transcriptase (Invitrogen, USA) according to the manufacturer’s instructions. The fragment of the 3HKT gene (from 329 to 648 in the CDS) was amplified via polymerase chain reaction (PCR) by using primers 3HKTRNAIF (5’-gagcgatcaatatggccaccc-3’) and 3HKTRNAIR (5’-aatgggcgttattccaggtgg-3’). PCR reactions were performed in a final volume of 25 μl containing 1 × PCR reaction buffer, 2 mM MgCl2, 0.4 μmol of each primer, 0.25 mM dNTPs, 1 μl of DMSO, 0.5 M betaine and 0.5 U Taq DNA polymerase (Promega, USA) according to the following program: 4 min at 95 ℃; 30 cycles of denaturation for 40 s at 95 ℃, annealing for 40 s at 58 ℃ and elongation for 20 s at 72 ℃; 10 min at 72 ℃. The amplified fragment was inserted into the pMD18-T plasmid construct to obtain pMD- 3HKT. The plasmid was sent to Shanghai Shengong Biotechnology Co., Ltd., for sequencing. The fragments were digested by HindIII/BamHI and XbaI/SalI and subsequently inserted into the corresponding cloning sites of plasmid T282 to acquire T282-3HKT. Both RNAi vector of pMaa7 IR/XIR and intermediate vector of T282-3HKT were digested by EcoRI and then ligated to produce the RNAi recombinant pMaa7 IR/3HKTIR [30].

### Algae transformation

The *Chlamydomonas* cells were grown to a density of 1–2 × 10^6^ cells ml^−1^. The cells were collected through centrifugation (10,000 × *g*, 3 min), washed twice and then resuspended in TAP medium (without arginine) to a cell density of approximately 1 × 10^8^ cells ml^−1^. The plasmid DNA was introduced into the cells using the glass bead method [31]. Briefly, 2 µg of plasmid DNA and 400 µl of cells were mixed with 100 µl of 20% polyethylene glycol (PEG) and 300 mg of sterile glass beads. The mixture was vortexed for 15 s, and the cells were washed from the glass beads and plated on TAP agar supplemented with 1.5 mM L-tryptophan, 5 µg paromomycin ml^−1^ and 5 µM 5-fluoroindole [32].

The *Chlorella* (3 × 10^7^ cells ml^−1^) was collected by centrifugation at 3000 g for 5 min. The cells were washed with liquid TAP medium and resuspended in electroporation buffer. For transformation, 2 µg plasmid DNA was added, and the mixture was incubated on ice for 15 min. The cell suspension was then transferred into an electrode cup and subjected to an electric shock in an electrometer (BTX, ECM 630, USA) with a 1600 V/cm voltage and a pulse time of 1 ms. Next, the suspension was transferred to TAP medium containing 60 mmol/l sorbitol and left to recover overnight. Finally, the cells were collected by centrifugation (10,000 g, 3 min) and plated on TAP agar medium plate containing 10 µg/ml paromomycin [33, 34].

### Mosquito feeding experiments

Mosquito feeding experiments using transgenic *Chlamydomonas*: the test mosquitoes were divided into the experimental and multiple control groups. Each group contained ten first-instar larvae. The larvae in the experimental groups were fed with the 3HKT RNAi transgenic *Chlamydomonas* strains (3HKT-A9, 3HKT-B1, 3HKT-B2, 3HKT-B5, 3HKT-B7, 3HKT-C2, 3HKT-C4, 3HKT-D2, 3HKT-D3 and 3HKT-D5), respectively, whereas those in the control groups were fed with non-transgenic *C. reinhardtii* CC425, water and fodder. Each experiment was repeated thrice. The mortality of the larvae was recorded daily. The surviving larvae were fed until the adult stage. The pupation time of the larvae was recorded. Some larvae were embedded in paraffin, sectioned, stained with hematoxylin-eosin (HE) and then examined under a light microscope (Nikon 200i). In the large-scale feeding experiment, more than 270 larvae hatched from eggs were selected for test treatment and controls. The duration of the experiment was 30 days. The larvae fed with the transgenic strain 3HKT-A9 were set as the test treatment, whereas the larvae fed with non-transgenic *C. reinhardtii* CC425 and fodder were used as controls. The survival numbers of larvae and adult mosquitoes were recorded. Mosquito-feeding experiments using transgenic *Chlorella*: The larvae in the experimental groups were fed with the 3HKT RNAi transgenic *Chlorella* strains (3HKT-1, 3HKT-3, 3HKT-12, 3HKT-21, 3HKT-26, 3HKT-33, 3HKT-37, 3HKT-38, 3HKT-51 and 3HKT-55), whereas those in the control group were fed with non-transgenic *Chlorella* HOC5, water and fodder. Each experiment was repeated thrice. The mortality of the larvae was recorded. To test the effect of transgenic *Chlorella* cell wall on the lethal effect of *Aedes* mosquitoes, we designed a feeding experiment in which the transgenic *C. vulgaris* were first frozen and thawed repeatedly in liquid nitrogen. Then, the *Chlorella* was collected by centrifugation, dissolved in water and fed to *Aedes* larvae. The larvae in the experimental groups were fed with the liquid nitrogen-treated or -untreated 3HKT RNAi transgenic *Chlorella* strains (3HKT-1, 3HKT-3 and 3HKT-12). The controls were fed with non-transgenic *Chlorella* HOC5, water and fodder.

### Detection of 3HKT gene expression in *Aedes* larvae

For qRT-PCR, 10–20 larvae from each treatment were collected and pooled together. Total RNA was isolated from the larvae using the TRIzol Reagent (Takara), according to the manufacturer’s instructions. Single-strand cDNA was synthesized from total RNA using oligo-dT primers with Bio-Rad iscript-selected cDNA synthesis kit. Real-time PCR was performed on a BioRad iCycler iQ Real-Time PCR Detection System using SYBR Green as a fluorescent dye. Primers used for RPS17 amplification were (5’-AAGAAGTGGCCATCATTCCA-3’) forward and (5’-GGTCTCCGGGTCGACTTC-3’) reverse. The primer sequences for 3HKT gene quantification were (5’-gagcgatcaatatggccaccc-3’) forward and (5’-aatgggcgttattccaggtgg-3’) reverse. The amplification rate of each transcript (Ct) was calculated using the PCR baseline subtracted method performed in the iCycler software at a constant fluorescence level. The cycle threshold (Ct) was determined in triplicate. Relative fold differences were calculated using the relative quantification analytical method (2^−ΔΔCT^) [35]. Expression levels of 3HKT were determined compared to the endogenous control, *Ae. aegypti* RPS17 (Ribo ribosomal protein S17, GenBank accession no. AAEL004175 [KY000705]) [36].

### Histological analysis and HE staining

The larvae were fixed for > 24 h, dehydrated with gradient alcohol and then embed in paraffin at 65 ℃ for 3 h. After that, the paraffin larvae were frozen at -20 ℃ and cut into 4-μm slices with a paraffin microtome, followed by drying at 60 ℃. For dewaxing, sections were treated with xylene for 20 min, 100% ethanol for 5 min and 75% ethanol for 5 min. Each section was stained with hematoxylin and eosin dye. Slides were observed using an Olympus BX53 microscope (Olympus, Tokyo, Japan).

### Statistical analyses

Data analysis was performed using SPSS. Data are presented as mean ± SD (standard deviation). Duncan’s multiple range tests and *t*-test were performed to examine significant differences between means. *P*-values < 0.05 were considered statistically significant. In all cases, asterisks indicate the statistical significance: *P < 0.05, **P < 0.01. Error bars show the standard deviation.

## Results

### 3HKT RNAi transgenic *Chlamydomonas* are lethal to* Ae. aegypti*

The target gene of this study is the 3HKT (GenBank: XM_021849682, AF435806) gene of *Ae. aegypti*, which has a full length CDS of 1203. The target region of RNAi silencing is between 329 and 648 in the CDS, which is located in the corresponding aminotransferase (class V) domain (from 20 to 382, Pfam 33.1 analysis results) (Additional file 1: Figure S1). Complementary DNA of *Ae. aegypti* was used as a template to amplify the 3HKT RNAi interference fragment, and a band of about 319 bp was obtained. This fragment was cloned into pMD18-T to obtain pMD-3HKT. Sequencing results showed that the cloned 3HKT fragment shared 100% homology with the *Ae. aegypti* 3HKT gene. After the recombinant RNAi vector of pMaa7IR/3HKTIR was transformed into *C. reinhardtii* CC425, 56 transformed algal strains were identified as positive by PCR and used for subsequent experiments. pMaa7IR/3HKTIR was also transformed into *Chlorella* HOC5 using the electro transformation method. A total of 73 transgenic *Chlorella* lines were identified as positive by PCR and used to perform subsequent experiments.

**Fig. 1:**
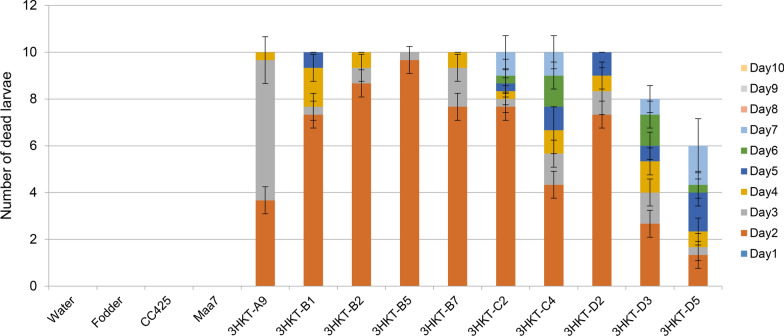
Mortality rate of *Aedes aegypti* larvae fed with 3HKT RNAi transgenic *Chlamydomonas*. Water: larvae fed with water; Fodder: larvae fed with fodder; CC425: larvae fed with non-transgenic *C. reinharditii* strain CC425; Maa7: larvae fed with empty plasmid Maa7IR/XIR transgenic *C. reinharditii* strain; 3HKT-A9 to 3HKT-D5: larvae fed with Maa7IR/3HKTIR transgenic *C. reinharditii* strains 3HKT-A9 to 3HKT-D5. Note: each treated or control group contained 10 *Aedes* larvae. Experiments were repeated thrice, and average values are reported. Duration: 10 days

The larvae fed with 3HKT RNAi transgenic *Chlamydomonas* began to die on the second day after feeding (3HKT-A9 to 3HKT-D5). Meanwhile, all the larvae fed with the transgenic *Chlamydomonas* 3HKT-B5 died on the third day, whereas those fed with strain 3HKT-A9 died on the fourth day. Except for the larvae fed with 3HKT-D3 and 3HKT-D5, all the larvae fed with transgenic *Chlamydomonas* died within 10 days. On the other hand, none of the larvae died in the control groups within 10 days, including the larvae fed with water, fodder, non-transgenic *Chlamydomonas* CC425 and empty plasmid Maa7IR/XIR transgenic *C. reinharditii* strain. These results indicated that oral feeding of 3HKT RNAi transgenic *Chlamydomonas* was lethal for *Aedes* larvae (Fig. 1).

**Fig. 2:**
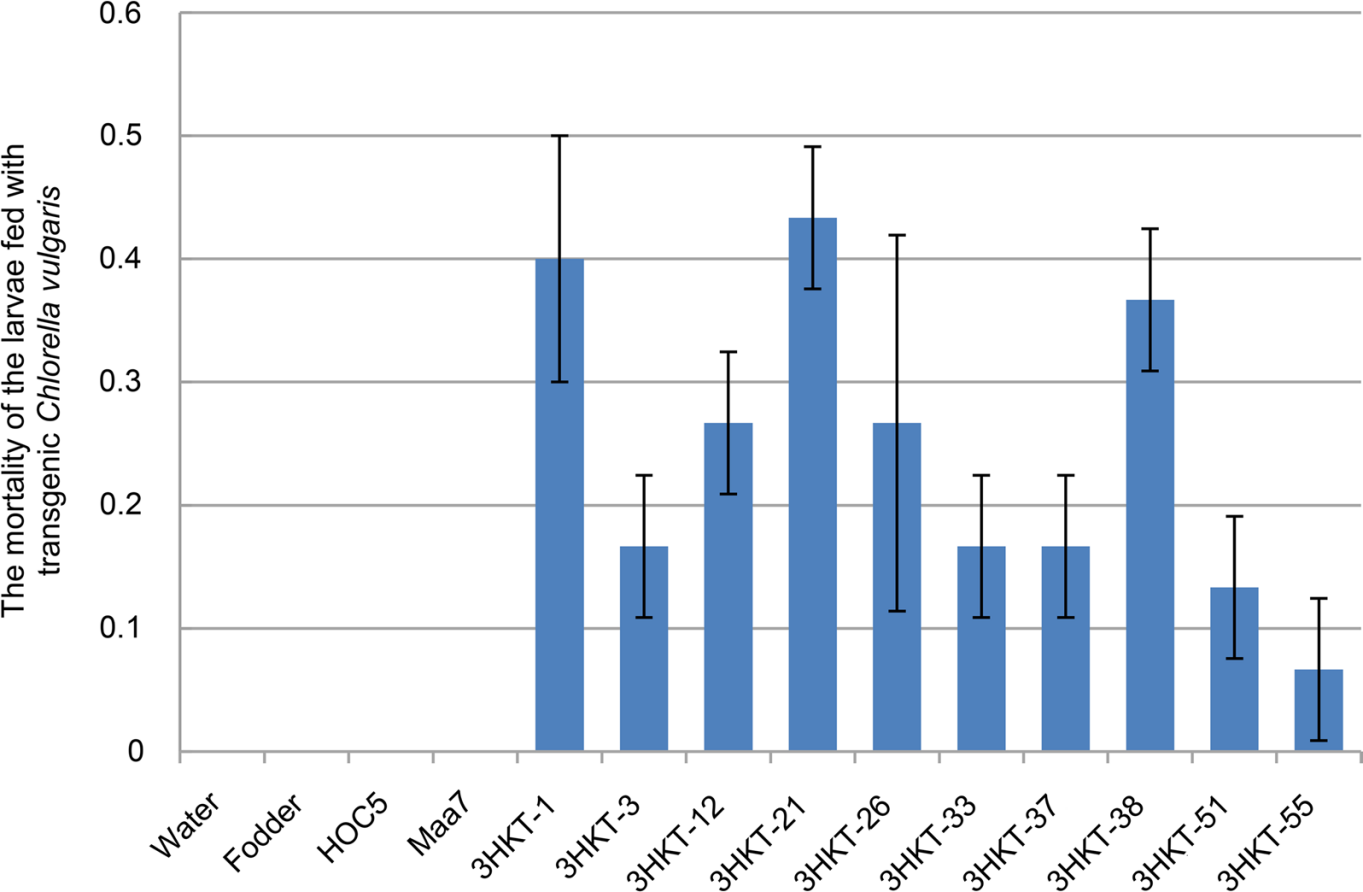
Mortality rate of *Aedes aegypti* larvae fed with transgenic *Chlorella vulgaris*. Water: larvae fed with water; Fodder: larvae fed with fodder; HOC5: larvae fed with non-transgenic *Chlorella vulgaris* strain HOC5; Maa7: larvae fed with empty plasmid Maa7IR/XIR transgenic *C. vulgaris* strain; 3HKT-1 to 3HKT-55: larvae fed with Maa7IR/3HKTIR transgenic *C. vulgaris* strains 3HKT-1 to 3HKT-55. Each treated or control group contained 10 *Aedes* larvae. Experiments were repeated thrice, and average values are reported. Duration: 20 days

The mortality rate of larvae fed with transgenic *Chlorella* was lower than for those fed with transgenic *Chlamydomonas*. The highest mortality rate of larvae fed with transgenic *C. vulgaris* was recorded in those fed with *Chlorella* 3HKT-21, with a mortality rate of 43% within 20 days. The lowest mortality rate (6.7%) was recorded in the larvae fed with 3HKT-55 transgenic *C. vulgaris* (Fig. 2). In contrast, the larvae fed with transgenic *Chlamydomonas* strains 3HKT-A9, 3HKT-B1, 3HKT-B2, 3HKT-B5, 3HKT-B7, 3HKT-C2, 3HKT-C4 and 3HKT-D2 had 100% mortality within 10 days, whereas the mortality of larvae fed with transgenic *Chlamydomonas* 3HKT-D3 and 3HKT-D5 were 80% and 60%, respectively (Fig. 1).

**Fig. 3:**
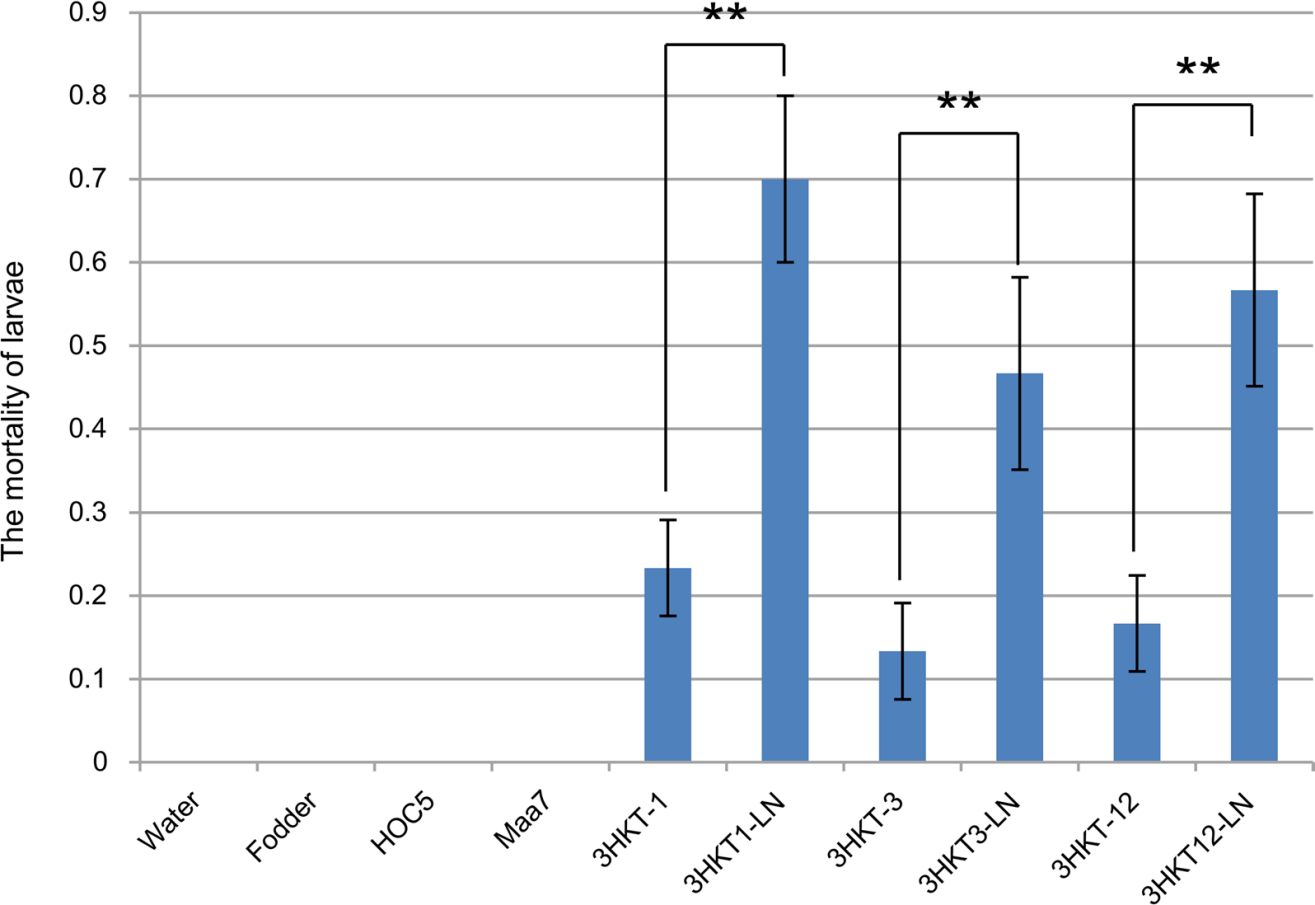
Mortality rate of *Aedes aegypti* larvae fed with transgenic *Chlorella vulgaris* following liquid nitrogen treatment. Water: larvae fed with water; Fodder: larvae fed with fodder; HOC5: larvae fed with non-transgenic *C. vulgaris* strain HOC5; Maa7: larvae fed with empty plasmid Maa7IR/XIR transgenic *C. vulgaris* strain; 3HKT-1, 3HKT-3 and 3HKT-12: larvae fed with transgenic *C. vulgaris* strains 3HKT-1, 3HKT-3 and 3HKT-12. 3HKT1-LN, 3HKT3-LN and 3HKT12-LN: larvae fed with liquid nitrogen treated transgenic *C. vulgaris* strains 3HKT-1, 3HKT-3 and 3HKT-12. Each treated or control group contained 10 *Aedes* larvae. Experiments were repeated thrice, and average values are reported. Duration: 10 days

To eliminate the influence of the cell wall, the repeated freezing and thawing method is used to break the cell wall of transgenic *Chlorella* so that the contents of the cell can play a role as far as possible. In this way, the mortality of larvae fed with liquid nitrogen-treated transgenic *Chlorella* 3HKT-1, 3HKT-3 and 3HKT-12 increased to 46.7%, 33.4% and 40%, respectively, 10 days after feeding. These results suggested that the cell wall of *Chlorella* might prevent 3HKT dsRNA from entering the digestive system of *Aedes* larvae (Fig. 3).

### The 3HKT mRNA levels in *Ae. aegypti* larvae fed with transgenic *algae*

**Fig. 4:**
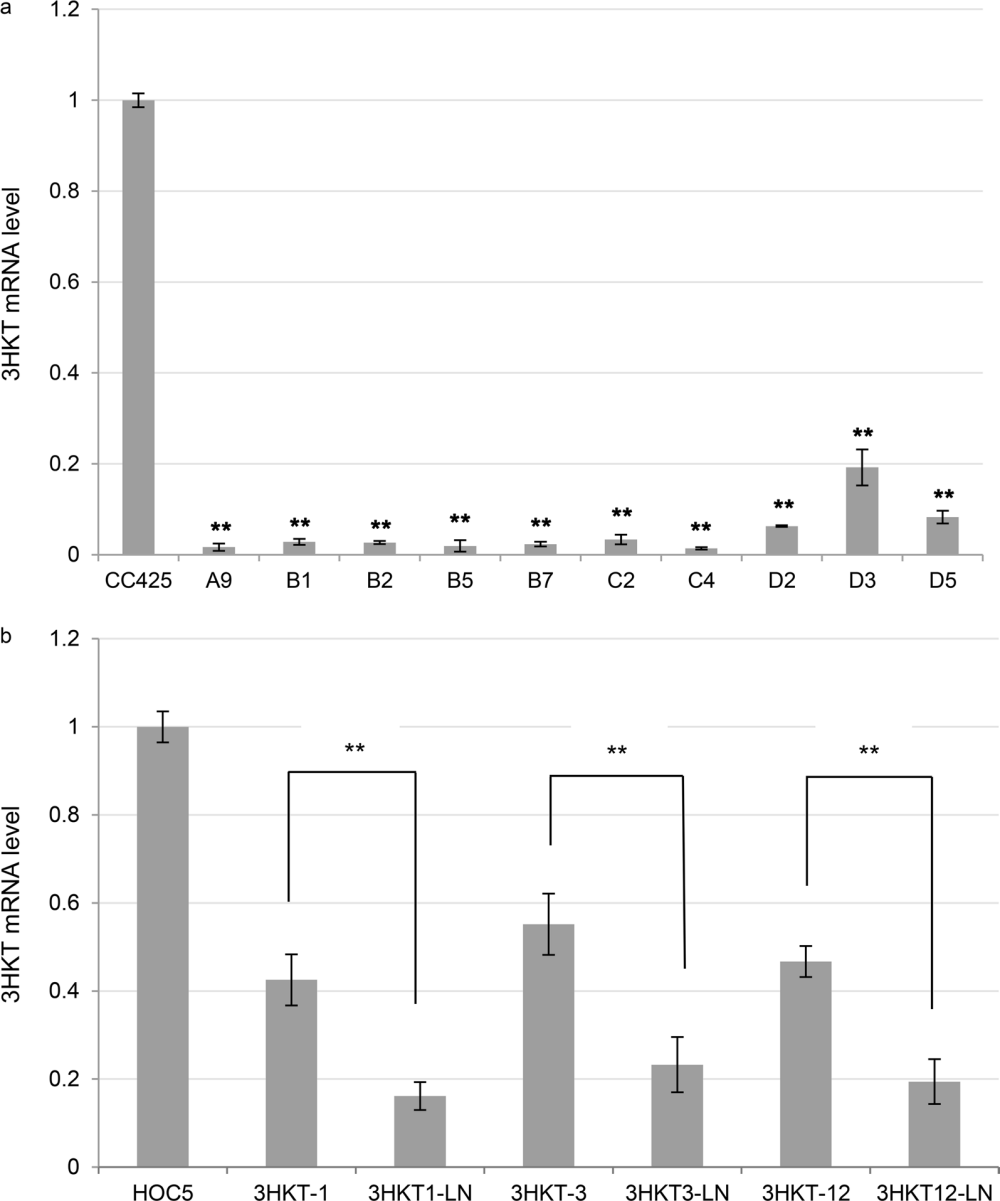
Relative 3HKT mRNA levels in *Aedes aegypti* larvae fed with transgenic algae. **a** 3HKT mRNA levels in larvae fed with transgenic *Chlamydomonas* strains after 5 days. CC425: larvae fed with non-transgenic *C. reinharditii* strain CC425; A9 to D5: larvae fed with transgenic *C. reinharditii* strains A9 to D5. **b** 3HKT mRNA levels in larvae fed with liquid nitrogen treated or not treated transgenic *Chlorella vulgaris* strains. HOC5: larvae fed with non-transgenic *C. vulgaris* strain HOC5; 3HKT-1, 3HKT-3 and 3HKT-12: Larvae fed with transgenic *C. vulgaris* strains 3HKT-1, 3HKT-3 and 3HKT-12. 3HKT1-LN, 3HKT3-LN and 3HKT12-LN: larvae fed with liquid nitrogen treated transgenic *C. vulgaris* strains 3HKT-1, 3HKT-3 and 3HKT-12

Real-time PCR was performed to examine the expression of the 3HKT gene in *Ae. aegypti* larvae fed with transgenic *Chlamydomonas*. Larvae fed with *C. reinhardtii* CC425 were used as control. The expression level of the 3HKT gene in the larvae fed with transgenic *Chlamydomonas* strain was significantly lower than in the control (Fig. 4a). The highest decrease (98.3%) in 3HKT gene expression was found in the larvae fed with the 3HKT-A9 strain. The 3HKT expression in other larvae fed with strains 3HKT-B1, 3HKT-B2, 3HKT-B5, 3HKT-B7, 3HKT-C2, 3HKT-C4, 3HKT-D2 and 3HKT-D5 reduced from 80.8% to 98.1%. These results showed that the transgenic *Chlamydomonas* could effectively silence the 3HKT gene in *Ae. aegypti*. In addition, qRT-PCR was also used to detect the 3HKT gene expression of the larvae fed with liquid nitrogen-treated transgenic *Chlorella*. Compared with fed untreated algae strains 3HKT-1, 3HKT-3 and 3HKT-12, larvae fed with the liquid nitrogen-treated strains 3HKT1-LN, 3HKT3-LN and 3HKT12-LN had significantly lower mRNA abundance (Fig. 4b).

### Feeding *Ae. aegypti* larvae with 3HKT RNAi transgenic *Chlamydomonas* led to tissue damage

We prepared paraffin sections of the larvae and performed microscopic observation to examine the changes in larvae tissues fed with 3HKT RNAi transgenic *Chlamydomonas*. Tissue sections showed that the larvae fed with *C. reinhardtii* CC425 had intact epidermis, thick, neatly arranged muscles and clear brush edges. Their midgut was normal, with an intact intestinal lumen (Additional file 2: Figure S2a–c). In contrast, the integumentary system of the larvae fed with transgenic *Chlamydomonas* was damaged, and their brush edge turned fuzzy. In addition, their muscles were unevenly distributed and disordered. The abnormal morphology of their midgut was characterized by an enlarged intestinal cavity (Additional file 2: Figure S2d–i).

### Large-scale feeding experiment

**Fig. 5:**
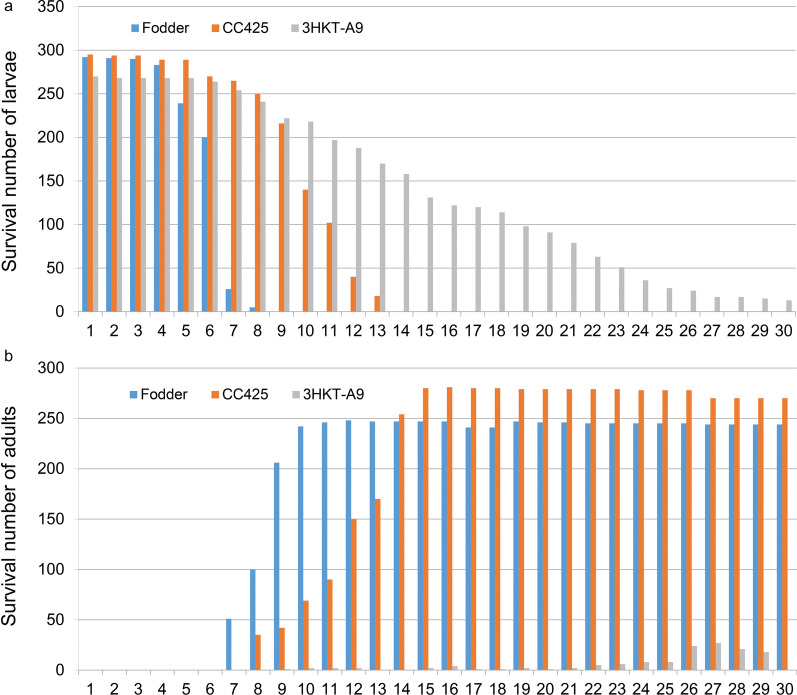
Pupation and survival rates of *Aedes aegypti* larvae (**a**) and adults (**b**) fed with 3HKT RNAi transgenic *Chlamydomonas*. Fodder, larvae fed with fodder; CC425, larvae fed with non-transgenic *C. reinhardtii* strain CC425; 3HKT-A9, larvae fed with 3HKT RNAi transgenic *C. reinhardtii* strain 3HKT-A9. Duration: 30 days

To test whether RNAi-based microalgae larvicides can suppress mosquito populations, approximately 300 larvae in each group were subjected to a large-scale breeding experiment for 30 days. The larvae fed with fodder began to pupate on the 4th day, and by day 8, all the larvae had pupated. The larvae fed with *C. reinhardtii* CC425 began to pupate on the 4th day, and by day 13, all the larvae had pupated. The larvae fed with transgenic *Chlamydomonas* began to pupate on the 6th day, and by day 30, 13 larvae had not pupated (Fig. 5a).

The survival rate of adult mosquitoes showed that the pupae of larvae fed with fodder fully emerged into adults on the 12th day, and the adult mosquitoes had a 30-day survival rate of 81.3%. The larvae fed with *C. reinhardtii* CC425 fully emerged into adults on the 16th day, and the adult mosquitoes had a 30-day survival rate of 90%. A total of 13 larvae that fed on transgenic *Chlamydomonas* had not pupated by day 30 after feeding. The survival rate was 0.0%, and this was exhibited by the uninterrupted death of larvae and adult mosquitoes (Fig. 5). The above results indicate that the 3HKT RNAi transgenic strain exerts obvious insecticidal effects and has potential for further field trials to control the population of *Aedes* mosquitoes.

## Discussion

Concerning public health, mosquitoes are the most harmful group of insects because they spread various pathogens and cause millions of deaths every year. Repeated use of a single compound insecticide leads to fragility and instability of the ecosystem and pesticide resistance. Thus, there is a need to develop alternative methods of controlling insect vectors [37]. Algae is an essential food source for many types of mosquito larvae, which feed on microbes, small aquatic animals (such as rotifers) and other tiny particles in the aquatic environment. The larvae can filter the algae in the water column, scrape the algae from the container or aquatic plant's surface, or scoop the algae from the bottom of the mosquito breeding aquatic habitat [38]. Some phytoplankton types are a source of nutritious food for mosquito larvae, whereas other phytoplankton types produce allelochemicals toxic to mosquitoes at different stages [38–42]. In nature, it is common for mosquito larvae to die before completing their development because of poisons from phytoplankton toxins or starvation while feeding on non-digestible phytoplankton [43, 44]. The use of microalgae to control mosquitoes is easy in the natural habitat and may not receive much opposition from the public. Besides, this technology is applied under strict adherence to government regulations regarding environmental and ecological security.

So far, only Kumar et al. [23] and our laboratory [26] have reported the use of RNAi microalgae to control mosquito populations by oral route. In their study, *Anopheles stephensi* larvae fed on the RNAi transgenic microalga showed reduced target gene expression levels and increased mortality rate of 30% to 53% compared to those fed wild-type algal cells [23]. In our previous study, silencing the HR3 gene of *Aedes* mosquitoes had some scale of lethal effect [26]. In this study, the mortality rate of *Aedes* larvae fed with transgenic *Chlamydomonas* ranged from 60 to 100% in small-scale tests and 100% in adult mosquitoes in large-scale feeding experiments. Moreover, we found that larvae fed with transgenic *Chlorella* exhibited a lower mortality rate (ranging from 6.7% to 43%), implying that *Chlamydomonas* was more effective than *Chlorella* in delivering dsRNA to the larvae. Based on the above findings, we speculated that transgenic recipient strain *C. reinhardtii* CC425, a cell wall-deficient algal strain, could release 3HKT RNAi molecules quickly out of the algae cells. These molecules then entered the midgut of *Aedes* mosquitoes, resulting in target gene silencing and insect death. Contrarily, *C. vulgaris* HOC5 with cell wall could slow down the release of 3HKT RNAi molecules, thus reducing the lethal effect on *Aedes* larvae. Given this, a new feeding method was implemented, in which the transgenic *Chlorella* were frozen and thawed repeatedly in liquid nitrogen to destruct the cell walls. In this way, the mortality of larvae fed with liquid nitrogen-treated transgenic *Chlorella* 3HKT-1, 3HKT-3 and 3HKT-12 increased from 33.4% to 46.7%. These results suggested that the cell wall of *Chlorella* might prevent 3HKT dsRNA from entering the digestive system of *Aedes* larvae (Fig. 3). However, compared with the model organism *C. reinhardtii*, the locally isolated wild species *C. vulgaris* HOC5 is more adapted to the natural habitat. Therefore, considering the ecological pressure in the field, the rapid release of dsRNA to kill *Aedes* mosquitoes may be more likely to cause mutations in *Aedes* mosquito. Therefore, although *Chlorella* has a lower lethality to *Aedes* mosquitoes, it is not easy to cause *Aedes* mutations and has a certain application value.

An interesting work was reported by Kang et al. [45] where expression of Cry proteins from *Bacillus thuringiensis* (Bt) in *Chlamydomonas* could kill mosquito larvae. They aimed to create a biological insecticide. Nevertheless, these proteins were bound to kill other non-target insects when released through genetically modified algae into the environment. By comparison, our study selected a specific region of *Aedes* 3HKT gene as the RNAi silencing region, which possessed species-specific characteristics. Theoretically, there is little toxicity to non-target organisms because RNAi binding and silencing are specific.

The use of biotechnology to control mosquitoes provides a new way to control mosquito populations. At present, the use of *Wolbachia* infection and gene splicing male infertility techniques are relatively successful cases [46–50]. The outdoor survival rate of the offspring of transgenic male sterile *Aedes* mosquito OX513A reached 15%–18% after eating tetracycline-containing animal food [51–53], which is undoubtedly a major environmental safety hazard. The obstacle to *Wolbachia* anti-mosquito technology is that *Wolbachia* can infect arthropod organisms, and there is a risk of gene drift [54–56].

We were devoted to controlling mosquito populations by releasing lethal algae to enclosed natural waters. Despite the need to face strict administrative approval and supervision, this approach is undoubtedly the cheapest and most environmentally friendly way to control mosquito populations. Future studies should focus on exploiting marker-free transgenic algae and locally available algae species, which can be used as transgenic receptors to maximize the environmental adaptability and to minimize adverse environmental impacts on other non-target organisms.

## Conclusions

3HKT RNAi transgenic algae are in some scales lethal to *Aedes aegypti*. The findings of this study indicate that technology based on microalgae RNAi might provide a new way to control mosquito populations.

## Supplementary Information

**Additional file 1: Figure S1.** Schematic diagram of coding region of 3-hydroxykynurenine transaminase (3HKT) in *Aedes aegypti*. The yellow part indicates the area silenced by RNAi, which is located in the 3HKT coding region between 329 and 648, in the corresponding aminotransferase (class V) domain.

**Additional file 2: Figure S2.** Tissue changes in *Aedes aegypti* larvae fed with 3HKT RNAi transgenic *Chlamydomonas*. a–c: The body of the larva fed with non-transgenic C. *reinharditii* strain CC425; d–i: The tissues of larva fed with 3HKT RNAi transgenic *Chlamydomonas* 3HKT-A9. Larvae fed with *C. reinhardtii* CC425 had intact epidermis, thick and neatly arranged muscles, and clear brush edges. Their midgut was normal, with an intact intestinal lumen (a–c). The integumentary system of the larvae fed with 3HKT RNAi transgenic *Chlamydomonas* was damaged, and their brush edge turned fuzzy. Their muscles were unevenly distributed and disordered. The midgut was characterized by an enlarged intestinal cavity (d–i).

## Data Availability

Data supporting the conclusions of this article are included in this published article and its additional files.

## Abbreviations

qRT-PCR: Quantitative reverse transcription-polymerase chain reaction
dsRNA: Double-stranded RNA
miRNA: MicroRNA
shRNA: Heterogeneous nuclear RNA
3-HKT: 3-Hydroxykynurenine transaminase
3-HK: 3-Hydroxykynurenine
XA: Xanthurenic acid
PEG: Polyethylene glycol
HE: Hematoxylin–eosin
