# Supplementary material for: Development of an RNAi-based microalgal larvicide for the control of Aedes aegypti
Source: Parasit Vectors. 2021 Aug 6;14:387. doi: 10.1186/s13071-021-04885-1 (PMC8344188; doi:10.1186/s13071-021-04885-1)

Additional file: **Figure S1.** The schematic diagram of coding region of 3-hydroxykynurenine transaminase (3HKT) in *Aedes aegypti*. The yellow part indicates the area silenced by RNAi, which is located in the 3HKT coding region between 329 and 648, in the corresponding aminotransferase (class V) domain.

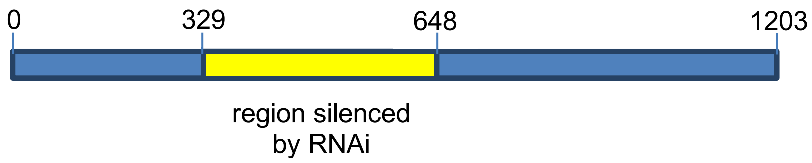

Supplement: Supplementary file 1 — Additional file 1: Figure S1. Schematic diagram of coding region of 3-hydroxykynurenine transaminase (3HKT) in Aedes aegypti. The yellow part indicates the area silenced by RNAi, which is located in the 3HKT coding region between 329 and 648, in the corresponding aminotransferase (class V) domain. [file 13071_2021_4885_MOESM1_ESM.pdf]
