# Supplementary material for: Development of an RNAi-based microalgal larvicide for the control of Aedes aegypti
Source: Parasit Vectors. 2021 Aug 6;14:387. doi: 10.1186/s13071-021-04885-1 (PMC8344188; doi:10.1186/s13071-021-04885-1)
Supplement: Supplementary file 2 — Additional file 2: Figure S2. Tissue changes in Aedes aegypti larvae fed with 3HKT RNAi transgenic Chlamydomonas. a–c: The body of the larva fed with non-transgenic C. reinharditii strain CC425; d–i: The tissues of larva fed with 3HKT RNAi transgenic Chlamydomonas 3HKT-A9. Larvae fed with C. reinhardtii CC425 had intact epidermis, thick and neatly arranged muscles, and clear brush edges. Their midgut was normal, with an intact intestinal lumen (a–c). The integumentary system of the larvae fed with 3HKT RNAi transgenic Chlamydomonas was damaged, and their brush edge turned fuzzy. Their muscles were unevenly distributed and disordered. The midgut was characterized by an enlarged intestinal cavity (d–i). [file 13071_2021_4885_MOESM2_ESM.pdf]

Additional file 2: Figure S2. Tissue changes in *Aedes aegypti* larvae fed with 3HKT RNAi transgenic *Chlamydomonas*. a, b, c: The body of the larva fed with non-transgenic *C. reinharditii* strain CC425; d-i: The tissues of larva fed with 3HKT RNAi transgenic *Chlamydomonas* 3HKT-A9. Larvae fed with *C. reinhardtii* CC425 had intact epidermis, thick and neat arranged muscles, and clear brush edge. Their midgut was normal, with an intact intestinal lumen (a, b, c). The integumentary system of the larvae fed with 3HKT RNAi transgenic *Chlamydomonas* was damaged, and their brush edge turned fuzzy. Their muscles were unevenly distributed, disordered. The midgut was characterized by enlarged intestinal cavity (d-i).

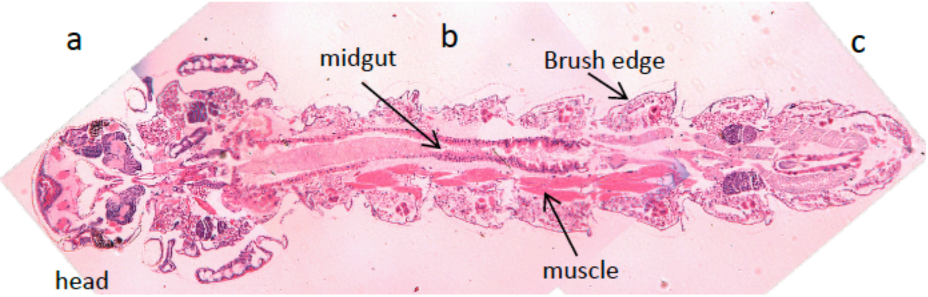
a
b
c
midgut
Brush edge
muscle
head

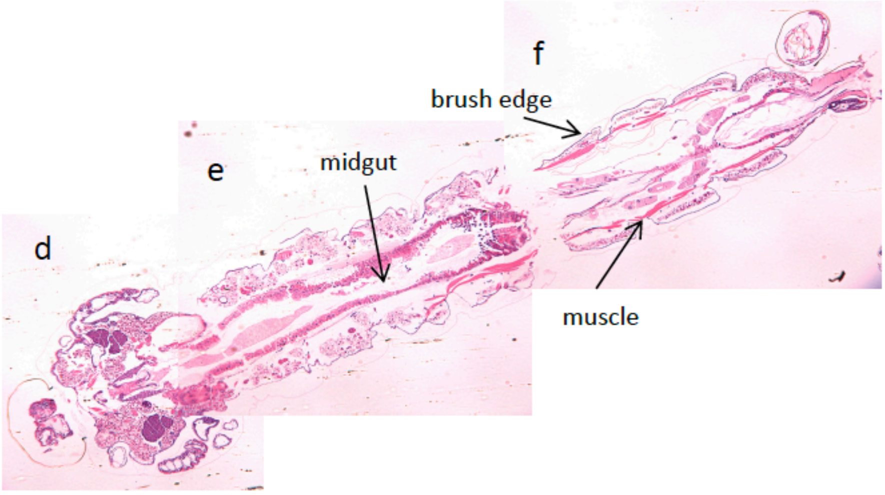
f
brush edge
e
midgut
d
muscle

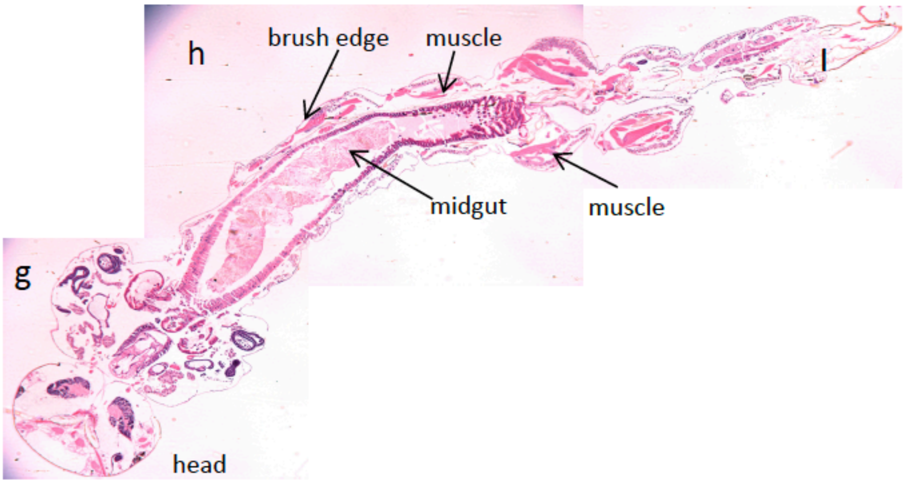
h
brush edge
muscle
I
midgut
muscle
g
head
